# Registration of Six Degrees of Freedom Data with Proper Handling of Positional and Rotational Noise

**DOI:** 10.6028/jres.118.013

**Published:** 2013-06-06

**Authors:** Marek Franaszek

**Affiliations:** National Institute of Standards and Technology, Gaithersburg, MD 20899

**Keywords:** 6DOF, noise, nonlinear least squares, pose determining systems, registration, robot-world/hand-eye calibration

## Abstract

When two six degrees of freedom (6DOF) datasets are registered, a transformation is sought that minimizes the misalignment between the two datasets. Commonly, the measure of misalignment is the sum of the positional and rotational components. This measure has a dimensional mismatch between the positional component (unbounded and having length units) and the rotational component (bounded and dimensionless). The mismatch can be formally corrected by dividing the positional component by some scale factor with units of length. However, the scale factor is set arbitrarily and, depending on its value, more or less importance is associated with the positional component relative to the rotational component. This may result in a poorer registration. In this paper, a new method is introduced that uses the same form of bounded, dimensionless measure of misalignment for both components. Numerical simulations with a wide range of variances of positional and rotational noise show that the transformation obtained by this method is very close to ground truth. Additionally, knowledge of the contribution of noise to the misalignment from individual components enables the formulation of a rational method to handle noise in 6DOF data.

## 1. Introduction

The pose of a three dimensional rigid body is determined by six degrees of freedom: three coordinates of the position vector (defining, e.g., the location of the center of mass) and three angles (e.g., Euler angles or yaw, pitch, and roll) which uniquely parameterize a 3×3 rotation matrix. A pose measuring instrument outputs position and orientation data in a coordinate frame defined by the pose of the instrument in a global coordinate system. Any data acquired by the instrument from two different locations need to be transformed into one coordinate system through a process known as registration. A similar procedure is required when a robot’s vision system collects data in one coordinate frame while the robot’s arm operates in another coordinate frame (robot-world/hand-eye calibration problem). Due to noise present in acquired 6DOF data (both in their positional and rotational parts) the alignment of two datasets is not perfect. Mathematically, both the registration and calibration problems can be formulated as the minimization of some kind of error measure *E*_pose_(***H***) where the homogeneous transformation ***H*** being sought consists of a rotation and a translation.

Various techniques have been developed to obtain ***H*** (see [1] for a comprehensive review). Some methods are based on iterative minimization [2,3], while others provide closed form solutions [4,5]. There are techniques that parameterize rotation with the help of quaternions [6,7]; others use an axis and angle representation [8]. Many approaches follow a separable solution strategy: first calculate the rotational part of ***H*** and then calculate the translation [9]. Fewer procedures simultaneously solve both the rotational and translational components of ***H*** [10,11]. Another class of techniques falls into a category of structure from motion with improved *L*_∞_ norm optimization [12,13], which enables recovery of a correct location scale [14].

Since 6DOF data contain both positional and rotational components, three different strategies of data handling are possible in the search for the best rotation: 1) use only the positional part of 6DOF data; 2) use only the rotational part; or 3) use both the positional and the rotational parts. The first method, 3DOF registration, is used in many applications where only 3D points are acquired [15–17]. Of course, whenever 6DOF data are available, the natural choice is to use all the data without wasting any part of them. However, there is a scaling problem associated with existing registrations based on full 6DOF data. The problem is inherent in the definition of the corresponding error function

(1) $$E_{\mathrm{pose}}=E_{\mathrm{rot}}+E_{\mathrm{loc}},$$

where *E*_rot_ is a dimensionless measure of error related to the rotational part of the data (three angles), while *E*_loc_ is the error caused by the positional part of data and is expressed in (length)^2^ units. This ill-defined expression is a consequence of using a homogenous matrix ***H*** and its Frobenius norm ║***H***║ as a measure of an overall error

(2) $$H=\left[\begin{array}{cc} \delta R_{3x3} & \delta v_{3x1}\\ 0_{1x3} & 1_{1x1} \end{array}\right]\;\mathrm{and}\;{\left\|H\right\|}^{2}=tr(H^{T}H),$$

where *δ****R*** is a 3x3 matrix related to the rotational part of 6DOF data, *δ****v*** is a vector related to the positional part of data, *tr*() is a trace of a matrix and ***H***^T^ is the matrix transpose of ***H***. Since *E*_rot_ is bounded while *E*_loc_ is not, changing length units (say, from mm to µm or to meters) can cause either a contribution of *E*_rot_ or *E*_loc_ to *E*_pose_ to be ignored. Some attempts to fix this problem are based on a heuristic scale factor *λ*

(3) $$E_{\mathrm{pose}}=E_{\mathrm{rot}}+\lambda E_{\mathrm{loc}}$$

with *λ* having a dimensionality of (length)^−2^. While introducing *λ* makes the expression for *E*_pose_ formally correct, this approach fails to resolve the main problem as it does not provide an objective method of setting a value of *λ*.

A self-adjusting weight approach was proposed for the first time in [18]. The error function was defined as

(4a) $$E_{\mathrm{pose}}=E_{\mathrm{rot}}/\sigma_{\mathrm{rot}}^{2}+E_{\mathrm{loc}}/\sigma_{\mathrm{loc}}^{2}$$

where $\sigma_{\mathrm{rot}}^{2}$ and $\sigma_{\mathrm{loc}}^{2}$ are the variances of the rotational and positional noise, respectively. Since both variances are usually not known in laboratory measurements, a multistep minimization of the error function defined in (3) was proposed with

(4b) $$\lambda \to \sigma_{\mathrm{rot}}^{2}/\sigma_{\mathrm{loc}}^{2}.$$

Initially, an arbitrary value was assigned to *λ* and the first minimization of *E*_pose_ was performed. The weight factor was then updated to *λ* = *E*_rot_/*E*_loc_ and substituted back into (3). Next, the new *E*_pose_ was minimized again and the whole process was repeated. Figure 2 in [18] shows that regardless of the initial value of *λ*, after only three steps, *λ* approaches a constant value, which is interpreted as the correct weight between the rotational and positional components of *E*_pose_. Unfortunately, this procedure removes only a mismatch in length units between *E*_loc_ and $\sigma_{\mathrm{loc}}^{2}$, so they both can be expressed in the same units, like mm or µm. The procedure does not provide a true measure of relative noise levels, because the limit in (4b) is equally as ill defined as *E*_pose_ in (1). Depending on the size of the bounding box containing positional data, the same positional noise *σ*_loc_ can be declared as large or small, regardless of the actual value of *σ*_rot_.

In this paper the mismatch between *E*_loc_ and *E*_rot_ in the registration procedure is removed in a different way. Instead of using the Euclidean norm to measure the distance between two sets of 3D points, *E*_loc_ is expressed as a sum of squared angular differences between matching vectors. Then, the same form of function can be used to calculate *E*_rot_. This formulation ensures that the ratio *E*_loc_/*E*_rot_ is truly a good measure of the relative amount of noise in the positional and rotational components of 6DOF data. Extensive numerical simulations reveal that it may be more advantageous to use only the positional or the rotational part of data in some experimental conditions.

## 2. Definition of Error Function

Two 6DOF datasets {***p****_j_*, ***A****_j_*} and {***q****_j_*, ***B****_j_*}, *j*=1,…,*N*, are acquired in two different coordinate systems where, for each *j*, ***p****_j_* and ***q****_j_* are two corresponding vectors and ***A****_j_* and ***B****_j_* are two corresponding 3x3 rotation matrices. The registration transformation consists of a rotation matrix ***R*** and a translation vector ***v***. In this paper, all rotation matrices are used in an axis and angle representation. The axis can be represented as a unit column vector ***u***

(5) $$u(\vartheta ,\varphi )={\left[\cos\vartheta \cos\varphi ,\cos\vartheta \sin\varphi ,\sin\vartheta \right]}^{T}$$

where (*ϑ*,*φ*) are elevation and azimuth angles, respectively. Then, the matrix of rotation ***R*** about axis ***u*** by angle *ρ* can be expressed by the Rodrigues formula

(6) $$R(u,\rho )=R(\vartheta ,\varphi ,\rho )=\cos\rho I+\sin\rho {\left[u\right]}_{s}+(1-\cos\rho )u⊗u$$

where ***I*** is 3x3 identity matrix and

(7) $${\left[u\right]}_{s}=\left[\begin{array}{ccc} 0 & -u_{z} & u_{y}\\ u_{z} & 0 & -u_{x}\\ -u_{y} & u_{x} & 0 \end{array}\right],u⊗u=uu^{T}.$$

Following separable procedures, the rotation ***R*** is found first and then the translation ***v*** is calculated as

(8) $$v=p_{\mathrm{ctr}}-Rq_{\mathrm{ctr}}$$

where ***p***_ctr_ and ***q***_ctr_ are centroids of *N* points {***p****_j_*} and {***q****_j_*}. So, the hard part of registration is to find a correct rotation ***R***. When only 3DOF positional data are available, the typical approach is to move the origins of coordinate systems to the corresponding centroids

(9) $${\bar{p}}_{j}=p_{j}-p_{\mathrm{ctr}},{\bar{q}}_{j}=q_{j}-q_{\mathrm{ctr}},$$

and then find a rotation ***R*** which minimizes the following Euclidean norm

(10) $$E_{\mathrm{loc}}(\vartheta ,\varphi ,\rho )=\frac{1}{N}\sum_{j=1}^{N}{\left\|{\bar{p}}_{j}-R(\vartheta ,\varphi ,\rho ){\bar{q}}_{j}\right\|}^{2}.$$

Such defined *E*_loc_ has dimensionality of (length)^2^ and causes problems when 6DOF data need to be registered. To avoid this problem, vectors ${\bar{p}}_{j}$ and ${\bar{q}}_{j}$ are normalized

(11) $${\tilde{p}}_{j}=\frac{p_{j}-p_{\mathrm{crt}}}{\left\|p_{j}-p_{\mathrm{ctr}}\right\|},\;{\tilde{q}}_{j}=\frac{q_{j}-q_{\mathrm{ctr}}}{\left\|q_{j}-q_{\mathrm{ctr}}\right\|}$$

and the error function is defined as

(12) $$E_{\mathrm{loc}}(\vartheta ,\varphi ,\rho )=1-\frac{1}{N}\sum_{j=1}^{N}w_{j}{\left({\tilde{p}}_{j}\cdot R(\vartheta ,\varphi ,\rho ){\tilde{q}}_{j}\right)}^{2}$$

where 0 ≤ *w_j_* ≤ 1 is a weight factor for a given *j*-th term and · stands for the dot product of two vectors. The error function so defined gauges the angular misalignment between vectors ${\tilde{p}}_{j}$ and $R{\tilde{q}}_{j}$. This error function can now be minimized to find the best rotation ***R*** by using any (perhaps gradient based) optimization procedure. The gradient of *E*_loc_ can be calculated as

(13) $$\frac{\partial }{\partial \gamma }E_{\mathrm{loc}}(\vartheta ,\varphi ,\rho )=-\frac{2}{N}\sum_{j=1}^{N}w_{j}({\tilde{p}}_{j}\cdot R{\tilde{q}}_{j})({\tilde{p}}_{j}\cdot R_{\gamma }{\tilde{q}}_{j})\mathrm{for}\;\gamma =\vartheta ,\varphi ,\rho$$

where individual partial derivatives of rotation matrix ***R****_γ_* are expressed as

(14a) $$R_{\vartheta }=\sin\rho {\left[\frac{\partial u}{\partial \vartheta }\right]}_{s}+(1-\cos\rho )\left(\frac{\partial u}{\partial \vartheta }⊗u+u⊗\frac{\partial u}{\partial \vartheta }\right)$$

(14b) $$R_{\varphi }=\sin\rho {\left[\frac{\partial u}{\partial \varphi }\right]}_{s}+(1-\cos\rho )\left(\frac{\partial u}{\partial \varphi }⊗u+u⊗\frac{\partial u}{\partial \varphi }\right)$$

(14c) $$R_{\rho }=-\sin\rho I+\cos\rho {\left[u\right]}_{s}+\sin\rho u⊗u$$

and derivatives of vector ***u*** can be explicitly evaluated from (5).

*E*_rot_ can be calculated using a similar form of error function as *E*_loc_ in (12). Since ***A****_j_* is the rotation matrix, its three columns are unit vectors, i.e., the first column ***A****_j_*(:,1) is a unit vector along the rotated *x* direction, the second column ***A****_j_*(:,2) is a unit vector along the rotated *y*, and ***A****_j_*(:,3) along the rotated *z* (and similarly for ***B****_j_*). Thus, *E*_rot_ can be defined as

(15) $$E_{\mathrm{rot}}(\vartheta ,\varphi ,\rho )=1-\frac{1}{3N}\sum_{j=1}^{N}\sum_{k=1}^{3}s_{j}(k){\left[A_{j}(:,k)\cdot R(\vartheta ,\varphi ,\rho )B_{j}(:,k)\right]}^{2}$$

where 0 ≤ *s_j_* (*k*) ≤ 1 is a weight factor for the corresponding (*j,k*) term. The gradient of *E*_rot_ can be calculated similarly as for *E*_loc_ in (13)

(16) $$\frac{\partial }{\partial \gamma }E_{\mathrm{rot}}(\vartheta ,\varphi ,\rho )=-\frac{2}{3N}\sum_{j=1}^{N}\sum_{k=1}^{3}s_{j}(k)[A_{j}(:,k)\cdot R\;B_{j}(:,k)][A_{j}(:,k)\cdot R_{\gamma }B_{j}(:,k)]\;\mathrm{for}\;\gamma =\vartheta ,\varphi ,\rho$$

with ***R****_γ_* calculated in (14). Finally, the full error function *E*_pose_ for registering 6DOF data using both the positional and rotational parts of data is defined as in (1), with *E*_loc_ defined by (12) and *E*_rot_ by (15). The gradient of *E*_pose_ can be calculated from gradients of *E*_loc_ and *E*_rot_ as defined in (13) and (16), respectively.

In order to use the above procedure, the weight factors *w_j_* in (12) and *s_j_* (*k*) in (15) must be defined as well as a starting point for minimization (*ϑ*_0_, *φ*_0_, *ρ*_0_). If there were no noise in the acquired rotational data ***A****_j_* and ***B****_j_*, then the following would hold for every *j*

(17) $$A_{j}=R\;B_{j}.$$

In reality, a slightly different matrix ***R****_j_* has to be calculated for each *j*

(18) $$R_{j}(u_{j},\rho_{j})=A_{j}B_{j}^{T}$$

where ***u****_j_* and *ρ_j_* are the axis and the angle of rotation and $B_{j}^{-1}=B_{j}^{T}$. Then, the normalized mean axis and the mean angle of rotation can be used as the starting point (*ϑ*_0_, *φ*_0_, *ρ*_0_) in a minimization of *E*_pose_

(19) $$u_{0}(\vartheta_{0},\varphi_{0})=\frac{\sum_{j=1}^{N}u_{j}}{\left\|\sum_{j=1}^{N}u_{j}\right\|}\;\mathrm{and}\;\rho_{0}=\frac{1}{N}\sum_{j=1}^{N}\rho_{j}$$

where ***u***_0_ depends on angles (*ϑ*_0_, *φ*_0_) as in (5). If the experimental rotational data ***A****_j_* and ***B****_j_* are not heavily affected by noise, then the starting rotation ***R***_0_ (*ϑ*_0_, *φ*_0_, *ρ*_0_) should be relatively close to the final best fit rotation R^*^ (ϑ^*^, φ^*^, ρ^*^). If only 3DOF positional data are available then ***R***_0_ can be easily constructed from two corresponding triplets of non-collinear data points.

Once the starting point for the minimization is determined, the weight factors *w_j_* in (12) and *s_j_* (*k*) in (15) can be calculated

(20a) $$w_{j}=1-0.5\left|u_{0}\cdot ({\tilde{p}}_{j}-{\tilde{q}}_{j})\right|,$$

(20b) $$s_{j}(k)=1-0.5\left|u_{0}\cdot (A_{j}(:,k)-B_{j}(:,k))\right|,\;\mathrm{for}\;k=1,2,3\;\mathrm{and}\;j=1,\ldots ,N,$$

where ${\tilde{p}}_{j}$ and ${\tilde{q}}_{j}$ are defined in (11) and ***A****_j_*(:,*k*) is the *k*-th column of data matrix ***A****_j_* (and similarly for ***B****_j_*). The rationale behind such defined weight factors is that the components of vectors ${\tilde{p}}_{j}$ and ${\tilde{q}}_{j}$ that are parallel to ***u***_0_ should be close to each other, and similarly for ***A****_j_* (:,*k*) and ***B****_j_* (:,*k*). If they are not, then a given *j*-th term is classified as an outlier and a small value is assigned to *w_j_* in (12) or *s_j_* (*k*) in (15), respectively.

## 3. Numerical Simulations

In order to test the performance of the introduced procedure, extensive numerical simulations were done. First, a primary 6DOF dataset was generated $\left[x_{j}^{A},y_{j}^{A},z_{j}^{A},\vartheta_{j}^{A},\varphi_{j}^{A},\rho_{j}^{A}\right]\;\mathrm{for}\;j=1,\ldots ,N$. For every *j*, the unit axis vector $u_{j}\left(\vartheta_{j}^{A},\varphi_{j}^{A}\right)$ was created as in (5) together with the corresponding rotation matrix $A_{j}(u_{j},\rho_{j}^{A})$ as in (6) and the position vector $p_{j}=\left[x_{j}^{A},y_{j}^{A},z_{j}^{A}\right]$. Then, a transformation was selected by setting the translation vector ***t***_0_ and three angles (*ϑ*_GT_, *φ*_GT_, *ρ*_GT_). The unit axis vector ***u***(*ϑ*_GT_, *φ*_GT_) was created as in (5) and the rotation matrix $\tilde{R}(-u,\rho_{\mathrm{GT}})$ was formed as in (6). Then, the secondary 6DOF dataset $\left[x_{j}^{B},y_{j}^{B},z_{j}^{B},\vartheta_{j,}^{B}\varphi_{j}^{B},\rho_{j}^{B}\right]$ could be created as follows. The position vector is $q_{j}=\left[x_{j}^{B},y_{j}^{B},z_{j}^{B}\right]$, where $q_{j}=\tilde{R}p_{j}+t_{0}+N(0,f)$. The positional noise is represented by ***N***(0, *f*), a vector with three components that are pseudo-random numbers obtained from a generator with Gaussian distribution, zero mean and standard deviation equal to *f*. The rotation matrix ***B****_j_* is calculated as

(21) $$B_{j}=\tilde{R}C_{j}\left(\vartheta_{j}^{A}+h\zeta_{j},\varphi_{j}^{A}+h\omega_{j},\rho_{j}^{A}+h\eta_{j}\right)$$

where the rotation matrix ***C****_j_* was calculated as in (6), *h* is a standard deviation of angular noise, and [ζ*_j_*, *ω_j_*, *η_j_*] are pseudo-random numbers obtained by the standard Gaussian generator (with zero mean and standard deviation equal to one). In order to make an easier comparison between the effects caused by angular noise (*h* in radians) and positional noise (*f* in mm), the standard deviation of positional noise was calculated as

(22) $$f=g\;L_{\mathrm{avg}}$$

where *g* is expressed in radians and *L*_avg_ is the averaged length of vectors ***p****_j_* centered at ***p***_ctr_

(23) $$L_{\mathrm{avg}}=\frac{1}{N}\sum_{j=1}^{N}\left\|p_{j}-p_{\mathrm{ctr}}\right\|.$$

Once a pair of primary and secondary data was generated, the best fit rotation R^*^(ϑ^*^,φ^*^,ρ^*^) was determined by three different methods. In method 1, only positional data were used and *E*_loc_, defined in (12), was minimized. In method 2, only rotational data were used and *E*_rot_, as defined in (15), was minimized. In method 3, full 6DOF data were used and *E*_pose_ was minimized. Each method yielded slightly different best fit rotations $R_{k}^{*}$ for *k* = 1, 2, 3. For each rotation, a deviation *d_k_* from a ground truth rotation ***R***_GT_ was calculated using the Frobenius norm (2)

(24) $$d_{k}=\left\|R_{k}^{*}-R_{\mathrm{GT}}\right\|/2\sqrt{2}\;\mathrm{for}\;k=1,2,3.$$

The matrix ***R***_GT_ is an inverse of the matrix $\tilde{R}(-u,\rho_{\mathrm{GT}})$ which was used to generate a secondary 6DOF data in (21), i.e.,

(25) $$R_{\mathrm{GT}}(\vartheta_{\mathrm{GT}},\varphi_{\mathrm{GT}},\rho_{\mathrm{GT}})={\tilde{R}}^{-1}(-u(\vartheta_{\mathrm{GT}},\varphi_{\mathrm{GT}}),\rho_{\mathrm{GT}})=\tilde{R}(u,\rho_{\mathrm{GT}}).$$

For every pair of noise parameters (*g*, *h*), the above procedure was repeated many times. First, in order to check the stability of results obtained for different noise realizations, for a given transformation $\left\{t_{0},\tilde{R}\right\}$ and primary data, *N*_noise_ secondary datasets were generated ${\left[x_{j}^{B},y_{j}^{B},z_{j}^{B},\vartheta_{j}^{B},\varphi_{j}^{B},\rho_{j}^{B}\right]}_{\left(n\right)}$, where *n* = 1,…, *N*_noise_. Each *n*-th dataset was obtained by using different sequences of random numbers [*ζ_j_*,*ω_j_*,*η_j_*] and ***N*** (0, *f*) for rotational and positional noise, respectively. In addition to this test, *M*_data_ primary datasets were generated ${\left[x_{j}^{A},y_{j}^{A},z_{j}^{A},\vartheta_{j}^{A},\varphi_{j}^{A},\rho_{j}^{A}\right]}_{\left(m\right)}$, where *m* = 1,…, *M*_data_. For each *m*-th primary dataset, *N*_noise_ secondary datasets were generated as described previously ${\left[x_{j}^{B},y_{j}^{B},z_{j}^{B},\vartheta_{j}^{B},\varphi_{j}^{B},\rho_{j}^{B}\right]}_{\left(n,m\right)}$. Finally, *K*_trans_ transformations were created ${\left\{t_{0},\tilde{R}\right\}}_{\left(k\right)}$ where *k* = 1,…, *K*_trans_. For each *k*-th transformation, each *m*-th primary dataset and each *n*-th random sequence, a secondary dataset ${\left[x_{j}^{B},y_{j}^{B},z_{j}^{B},\vartheta_{j}^{B},\varphi_{j}^{B},\rho_{j}^{B}\right]}_{\left(n,m,k\right)}$ was generated. Overall, for every pair of noise parameters (*g*, *h*), a total of *N*_tot_ = *N*_noise_ x *M*_data_ x *K*_trans_ secondary datasets were generated. Each secondary dataset was registered to its corresponding primary data using each of the three different methods described earlier.

All numerical calculations were performed on a 32-bit PC in double precision. A standard quasi-Newton minimization algorithm as implemented by Davidon-Fletcher-Powell (DFP) in [19] was used in all optimizations. Exit criteria from this iterative procedure were set to 10^−6^ for both the minimum change in step and the flatness of a gradient. Pseudo-random numbers were generated using the *gasdev* function provided in [19].

## 4. Results

**Fig. 1:**
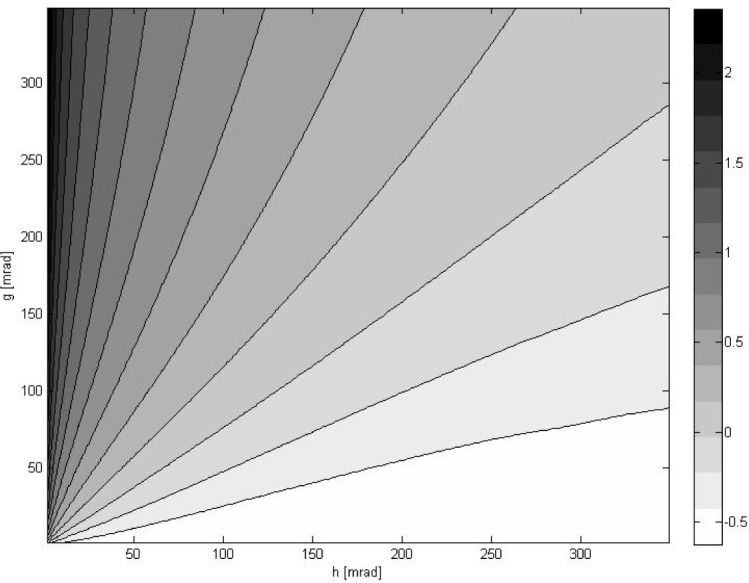
Contour plot of the mean ratio $\bar{\alpha }$ defined in (26) on a logarithmic scale as a function of a standard deviation of positional noise *g* and rotational noise *h*. For easier interpretation, both noise parameters are shown in milliradians. In simulations *g* was converted to millimeters using (22).

**Fig. 2:**
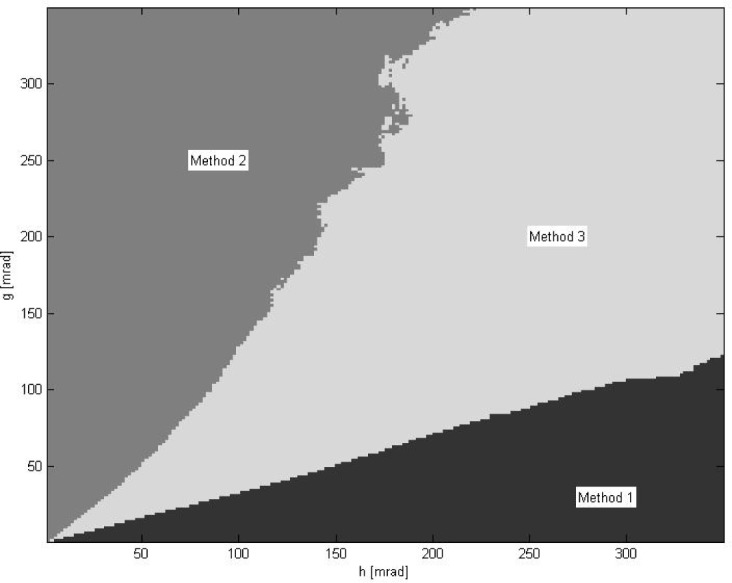
The diagram showing which of the three methods delivered most frequently the best registration. In method 1, *E*_loc_ was minimized using only the positional data; in method 2, *E*_rot_ was minimized using only the rotational data; in method 3, *E*_pose_ was minimized using full 6DOF data. For each pair of noise parameters (*g*, *h*), a total of *N*_tot_ = 1,600 registrations of different data pairs were performed.

**Fig. 3:**
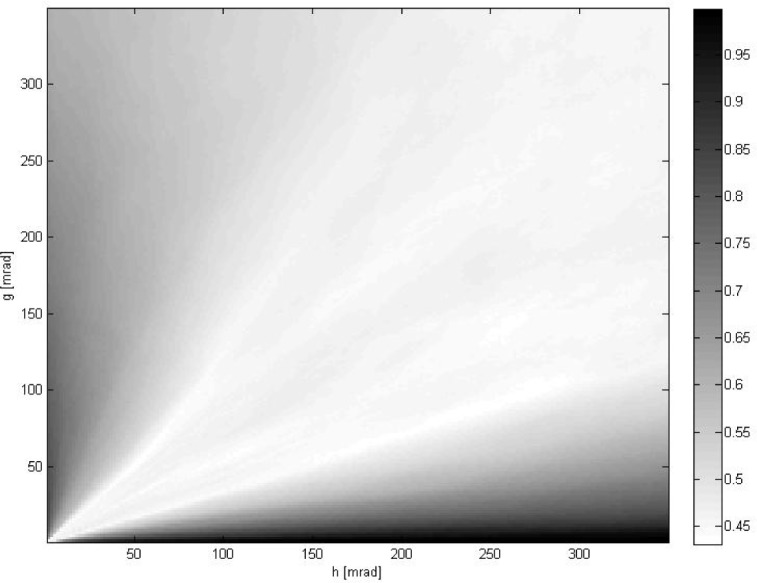
The frequency of winning by the best method shown in Fig. 2 vs. positional and rotational noise parameters (*g*, *h*).

**Fig. 4:**
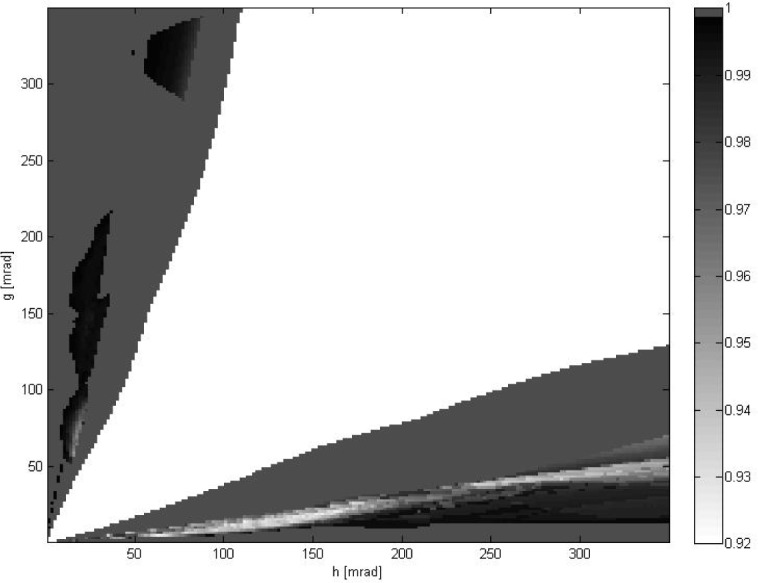
The frequency of correct predictions vs. noise parameters (*g*, *h*). Based on a value of *E*_loc_ / *E*_rot_, the two best methods are predicted. The central white area corresponds to the inconclusive region where no prediction could be made because *E*_loc_ ≈ *E*_rot_. Note that the value of color on the color bar for the highest frequency 1.0 deviates intentionally from a linear scale for better visualization.

**Fig. 5:**
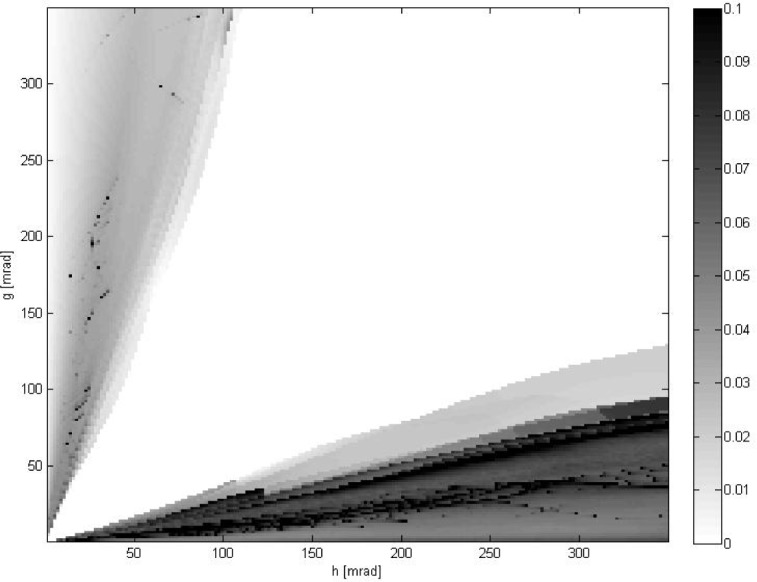
The mean ratio $\bar{\kappa }$ defined in (27) vs. noise parameters (*g*, *h*). As in Fig. 4, the central white part of the plot corresponds to an inconclusive region on (*g*, *h*) plane.

Figures 1–5 show the results of simulations obtained for 200 × 200 pairs of noise parameters (*g*, *h*). On average, 5–9 iterative steps were needed for the DFP minimization procedure to converge. The length of each dataset (primary or secondary) is *N* = 10, *N*_noise_ = 16, *M*_data_ = 10, *K*_trans_ = 10, so there are *N*_tot_ = 1,600 pairs of (primary, secondary) data requiring registration for each (*g*, *h*). Figure 1 shows the mean ratio $\bar{\alpha }$ averaged over all *N*_tot_ cases and displayed in a logarithmic scale

(26) $$\bar{\alpha }=\frac{1}{N_{\mathrm{tot}}}\sum_{l=1}^{N_{\mathrm{tot}}}\alpha_{l},\;\alpha_{l}=\frac{E_{\mathrm{loc}}\left(v_{\mathrm{pose}}^{\left(l\right)}\right)}{E_{\mathrm{rot}}\left(v_{\mathrm{pose}}^{\left(l\right)}\right)},$$

where $v_{\mathrm{pose}}^{\left(l\right)}$ is the solution obtained by minimizing *E*_pose_ for the *l*-th pair of data. Figure 2 shows which method most frequently delivered the smallest deviation from ground truth *d_k_* as defined in (24). Figure 3 displays how often a given method delivered the best results.

Figure 4 shows the outcome of a prediction procedure defined as follows. For each pair of noise parameters (*g*, *h*) and each *l*-th pair of data, the ratio *α_l_* was calculated as in (26). Then, if *α_l_* ≤ *T*_low_ or *α_l_* ≥ *T*_high_, where *T*_low_ < *T*_high_ are predefined parameters, the prediction was made. If *α_l_* ≥ *T*_high_, then the best results were expected to be delivered either by method 2 (minimization of *E*_rot_, positional data ignored) or by method 3 (minimization of *E*_pose_, full 6DOF data used). Similarly, if *α_l_* ≤ *T*_low_, then the best results were expected from either method 1 (minimization of *E*_loc_, rotational data ignored) or from method 3. This prediction was compared with the actual deviations from ground truth *d_k_*, *k* = 1,2,3. If the prediction was correct, the number of successful predictions for that pair (*g*, *h*) was increased by one. This process was repeated for every *l*-th pair of data (*l* = 1,…, *N*_tot_). Displayed in Fig. 4 is the number of successful predictions divided by *L*, where *L* is the total number of cases for which *α_l_* ≤ *T*_low_ (or *α_l_* ≥ *T*_high_) for a given (*g*, *h*). The central white part of the plot corresponds to an inconclusive region in (*g*, *h*) where no prediction could be made, because *T*_low_ <*α_l_* < *T*_high_, i.e., *L* = 0. The results presented were obtained for *T*_low_ = (1/3)^2^ and *T*_high_ = 3^2^. Figure 5 shows the mean ratio $\bar{\kappa }$ vs. noise parameters (*g*, *h*)

(27) $$\bar{\kappa }=\frac{1}{L}\sum_{l=1}^{L}\kappa_{l},\kappa_{l}=\Delta_{\mathrm{best}}^{\left(l\right)}/\Delta_{\mathrm{tot}}^{\left(l\right)},$$

where $\Delta_{\mathrm{best}}^{\left(l\right)}$ is the difference between the two smallest deviations *d_k1_* and *d_k2_* obtained with the predicted two best methods *k1* and *k2* applied to *l*-th data pair, and $\Delta_{\mathrm{tot}}^{\left(l\right)}$ is the difference between the largest and the smallest deviation for the same *l*-th pair. Similarly as in Fig. 4, the central white part of the plot corresponds to an inconclusive region in (*g*, *h*) where *L* = 0.

## 5. Discussion

The results presented in Fig. 1 indicate that, as expected, the ratio $\bar{\alpha }$ correlates well with the ratio of noise parameters *g*/*h*. This is important because the systems that are used for pose determination usually do not provide much information about the noise levels present in positional and rotational data. When the ratio $\bar{\alpha }$ becomes small, one may expect that discarding the rotational part of 6DOF data may, on average, lead to better registration. Similarly, for $\bar{\alpha }$ large, discarding the positional part of the full data may on average yield a better result. For intermediate values of $\bar{\alpha }$ using full 6DOF data should lead to the best results. Figure 2 and 3 confirm that these intuitive expectations are indeed correct.

For small or large values of $\bar{\alpha }(\bar{\alpha }\leq T_{\mathrm{low}}\;\mathrm{or}\;\bar{\alpha }\geq T_{\mathrm{high}})$, the procedure introduced in this paper predicts very well which of the three registration methods provides the best results. The data shown in Fig. 4 indicate that the highest ratio of false predictions does not exceed 0.08 (in a region of small $\bar{\alpha }\leq T_{\mathrm{low}}$, around noise parameters (*g*, *h*) ≈ (15, 120)). For the majority of (*g*, *h*) where either $\bar{\alpha }\leq T_{\mathrm{low}}$ or $\bar{\alpha }\geq T_{\mathrm{high}}$, the prediction rate is exactly 1 or very close to 1.

Overall, the data shown in Figs. 2–5 confirm that minimization of *E*_pose_ (the third method, full 6DOF data used) yields consistently good results in a whole range of investigated noise parameters (*g*, *h*). The third method most frequently delivers the best or second best result. In the latter case, for $\bar{\alpha }\leq T_{\mathrm{low}}$ or $\bar{\alpha }\geq T_{\mathrm{high}}$, the difference between the two best methods is in most cases two orders of magnitude smaller than the difference between the worst and the best method; see Fig. 5. This means there is a large difference between the worst method and the remaining two methods. At the same time, differences between the two best methods are small. Thus, using full 6DOF data and minimizing *E*_pose_ seems to be the best or close to the best strategy across the whole range of noise parameters (*g*, *h*). For very small or very large values of the ratio *E*_loc_ (**v**_pose_)/*E*_rot_ (**v**_pose_), it may be worthwhile to discard the noisy part of the 6DOF data and to redo the minimization using *E*_loc_ or *E*_rot_ with only positional or rotational data, respectively. A similar conclusion was formulated already in [3] without a systematic study of the mutual relation between positional and rotational noise. That observation was based on particular parameters used in the simulations: length of position vector ║***p****_j_*║ ∈ [500 mm, 1000 mm], length of translation ║***t***_0_║ = 800 mm, positional noise 1 mm and rotational noise 2.5 mrad (using a uniform random number generator). However, during the simulations, ║***t***_0_║ and ║***p****_j_*║ were calculated in meters. Only positional data were also used in another hand-eye calibration procedure. The minimum variance method introduced in [20] delivered better results than two other methods [8,18]. Systematic studies presented in this paper explain why this apparently surprising conclusion can be correct.

One may wonder why for small or large values of $\bar{\alpha }$ the predictions are not perfect, as the data in Fig. 4 indicate. However, it should be remembered that a residual value of the error function (like *E*_rot_ or *E*_loc_) provides only an indication of the noise level present in experimental data, not the actual deviation of best fit parameters from the unknown ground truth.

## 6. Conclusions

Performance of the iterative minimization procedure for registration of 6DOF noisy data was studied in computer simulations. The properly defined error function *E*_pose_ removed the mismatch between the positional component of the error (unbounded, in length units) and the bounded, dimensionless rotational component. The error function *E*_pose_ can be minimized and the resulting rotation matrix provides a good approximation to the true rotation across a large range of positional and rotational noise variances. Thus, both the developers and the users of pose determining systems could benefit from being able to properly gauge a relative amount of noise in the positional and the rotational parts of 6DOF data.
